# Gum Chewing Inhibits the Sensory Processing and the Propagation of Stress-Related Information in a Brain Network

**DOI:** 10.1371/journal.pone.0057111

**Published:** 2013-04-03

**Authors:** Hongbo Yu, Xi Chen, Jinting Liu, Xiaolin Zhou

**Affiliations:** 1 Center for Brain and Cognitive Sciences and Department of Psychology, Peking University, Beijing, China; 2 Key Laboratory of Machine Perception (Ministry of Education), Peking University, Beijing, China; West China Hospital of Sichuan University, China

## Abstract

Stress is prevalent in human life and threatens both physical and mental health; stress coping is thus of adaptive value for individual's survival and well-being. Although there has been extensive research on how the neural and physiological systems respond to stressful stimulation, relatively little is known about how the brain dynamically copes with stress evoked by this stimulation. Here we investigated how stress is relieved by a popular coping behavior, namely, gum chewing. In an fMRI study, we used loud noise as an acute stressor and asked participants to rate their feeling of stress in gum-chewing and no-chewing conditions. The participants generally felt more stressful when hearing noise, but less so when they were simultaneously chewing gum. The bilateral superior temporal sulcus (STS) and the left anterior insula (AI) were activated by noise, and their activations showed a positive correlation with the self-reported feeling of stress. Critically, gum chewing significantly reduced the noise-induced activation in these areas. Psychophysiological interaction (PPI) analysis showed that the functional connectivity between the left AI and the dorsal anterior cingulate cortex (dACC) was increased by noise to a lesser extent when the participants were chewing gum than when not chewing gum. Dynamic causality modeling (DCM) demonstrated that gum chewing inhibited the connectivity from the STS to the left AI. These findings demonstrate that gum chewing relieves stress by attenuating the sensory processing of external stressor and by inhibiting the propagation of stress-related information in the brain stress network.

## Introduction

Stress, referring to the consequence of an organism's failure to respond adequately to physical or psychological demands [1], is common in modern society. Such demands include, for example, exposure to unpleasant temperature or noise, and preparing for an important job interview. Chronic exposure to stress is detrimental to physical and mental health [2]–[4] and may ultimately lead to diseases [5], [6]. On a shorter time scale, stress elicits a host of neural and endocrine responses, characterized by the activation of the hypothalamic-pituitary-adrenal (HPA) axis and the sympathetic nervous system, which in turn results in increased corticosteroids level, heart rate, and skin conductance [7].

Recent neuroimaging studies, by testing healthy human subjects and their physiological responses to stressful stimuli/events (i.e., the stressor), have identified a number of brain areas responsive to stress, including the anterior cingulate cortex (ACC) and the insula [8]–[11]. For instance, Gianaros et al. [9] asked participants to perform a demanding cognitive task (e.g., the Stroop task), which was effective in inducing stress, while measuring their blood pressure and brain metabolic signal via functional magnetic resonance imaging (fMRI). They found that the stress-induced decrease of the capacity of the arterial baroreflex for control short-term fluctuations in blood pressure was accompanied by greater activity in the ACC, the insula, and the amygdala. These areas are known to be responsible for cardiovascular control. Importantly, the functional connectivity between the ACC and the insula was higher in the stressful situation [9], indicating enhanced adjustment of the stressor-evoked cardiovascular changes. Similarly, Hermans et al [10] found that viewing aversive, stress-inducing movies enhanced the activity and the interconnectivity within a brain network, which included the ACC and the insula. Moreover, the insula and the ACC are found to be co-activated in phobia sufferers both when undergoing phobic symptom provocation and when attending their own heart beat, suggesting that these two brain areas are associated with the integration of perceived stimulus characteristics and bodily responses ultimately leading to conscious feelings [12]. This is consistent with the view that the insula is the center for “interoception”, i.e., to perceive the bodily states (e.g., temperature, blood pressure and etc.,) and to transform the otherwise unconscious physiological responses to awareness and feelings [13]–[17].

Interoceptive stimuli are those of high biological salience to the organism, such as thirst, dyspnea (or ‘air hunger’), sexual arousal, heartbeat, and etc [15]. The presence of such events alters the physiological states of the organism by inducing anxiety, excitement, and stress, thereby calling for control of the autonomic responses to the low-level physiological challenge [16]. The rostral part of the cingulate cortex has been consistently implicated in emotion regulation [18]–[21]. Thus, it is conceivable that the ACC integrates afferent information received from, for example the insula, signaling the presence of a stressor (e.g., unpleasant temperature or a state of hypoglycemia), and prepares the organism for the potential challenge [9]. Indeed, several studies have reported the co-activation or increased functional connectivity between the AI and the ACC in participants facing stress-induced cognitive tasks [9], viewing aversive stimuli (10), or undergoing phobic symptom provocation [12].

When facing stress in daily life, individuals may adopt different approaches to cope with stress [22]. In some cultures, one popular means to mitigate stress is to chew gum. The original work by Hollingworth demonstrated that gum chewing reduces tension and “the surplus energy … goes unwittingly to the main work” [23]. Recent psychological and physiological studies support and extend what Hollingworth found more than half a century ago, showing that gum chewing can relieve mental stress and improve task performance [24]–[27]. For instance, in Scholey et al. [25], the participants were asked to perform on the Multi-tasking module in which the participants had to carry out four cognitive tasks simultaneously, including mental arithmetic, Stroop task, memory search and visual monitoring. The participants underwent this 20-min multi-task module three times on each of the two experiment days. The first session established baseline performance and stress reactivity on that day. The other two sessions were performed under a chewing and a no chewing condition. Both self-reports (State-Trait Anxiety Inventory, STAI; Stress visual analogue scale, SVAS) and stress-specific physiological index (salivary cortisol level) showed the effectiveness of the tasks in inducing stress and anxiety, indicated by the higher STAI and SVAS ratings and higher cortisol level after performing the baseline session as compared with the data collected before the session. Importantly, gum chewing significantly reduced the task-induced stress indicated both by the behavioral and by the physiological measures. Based on these findings, two kinds of mechanism have been proposed [24]–[27]: on the one hand, gum chewing improves attention and cognitive function, thus helps to ignore external stressor and improve performance on main tasks; on the other hand, gum chewing has positive effects on mood in face of stressors, whereby the experienced stress is (partially) canceled out. These two mechanisms are not exclusive to each other; rather, they might work in concert to reduce the feeling of stress.

**Figure 1:**
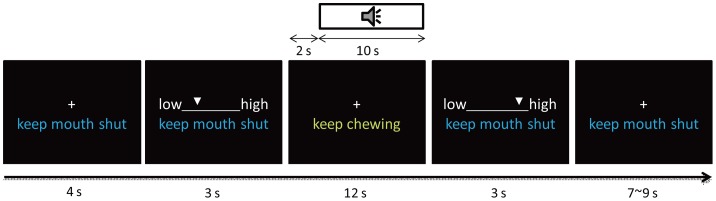
Sequence of events in a trial for functional MRI scanning.

Nevertheless, the brain basis of gum chewing as a way of stress coping has not been investigated directly. Here, we presented the participants with unpleasant noise, which is an effective elicitor of stress [28], and recorded the participants' hemodynamic responses in gum-chewing and no-chewing conditions. During a 30 s-trial (see Figure 1), participants were asked to rate their experienced stress on the stress visual analogue scale (SVAS), before (at *time*  = 5 s, referred to as SVAS-5 subsequently) and after (at *time*  = 20 s, SVAS-20) the Chew/Noise period. Thus, participants underwent four conditions: NoChew_NoNoise, NoChew_Noise, Chew_NoNoise, Chew_Noise. Using fMRI and connectivity algorithms (e.g., the psychophysiological interaction, PPI, and the dynamic causality modeling, DCM), we aimed to 1) identify the brain network for processing and regulating noise-induced stress and 2) examine how does gum chewing interfere with stress processing and relieve stress. On the basis of the previous findings concerning the neural processing of stressful stimulation [8]–[11] and the functions of the insula and the ACC in representing and regulating interoceptive challenges [13]–[17], we predicted that the anterior insula (AI) would be activated by noise-induced stress, and that the AI and the ACC would be more strongly connected when the noise is present than when it is absent. Gum chewing may relieve stress by dampening the sensory processing of noise, by inhibiting the propagation of stress-related information within the stress related network, or both.

## Result

### Behavioral results

**Figure 2:**
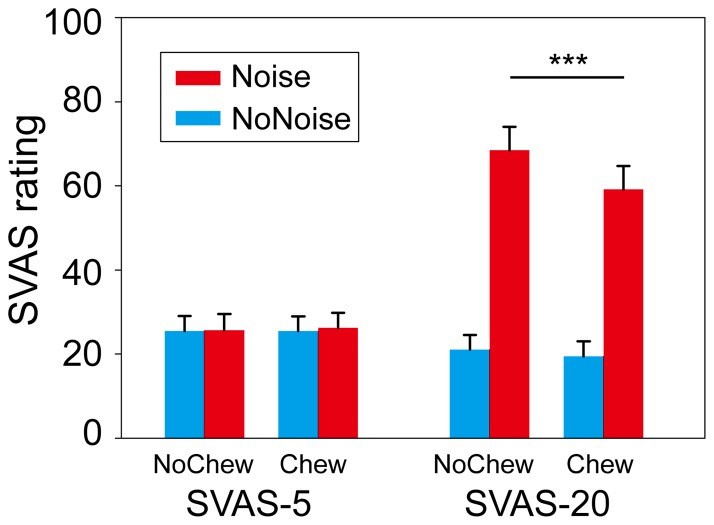
Experienced stress before the presentation of noise (SVAS-5, left) and after the presentation of noise (SVAS-20, right) as a function of noise presentation and gum chewing in the fMRI session. * *p*<0.05.

For the SVAS-5 rating, no effect reached significance, indicating that the baseline stress state was equivalent across the four conditions. For the SVAS-20 rating, both the main effects and the interaction were significant (Figure 2, see supporting File S1 for details). The noise-induced stress was lower in the Chew (*M* = 45, *SD*  = 18) than in the NoChew condition (*M* = 56, *SD*  = 20), *t*(15)  = 2.61, *P*<0.05.

### FMRI Results

#### Factorial model

**Figure 3:**
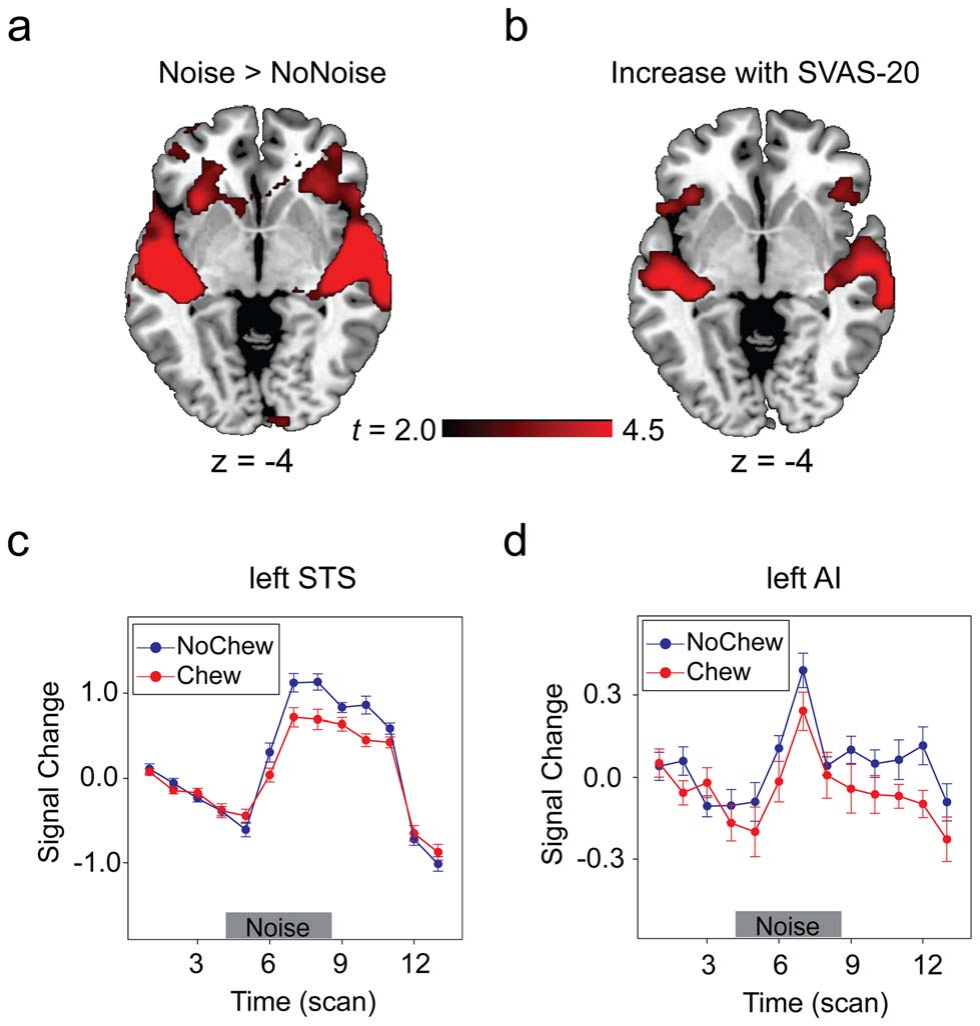
Brain regions revealed by the factorial and parametric models. (A) Brain regions sensitive to noise and noise-induced stress (“Noise > NoNoise”). (B) Brain regions in which the activation level positively correlates with the ratings of the subjectively experienced level of stress. (C) and (D) The time course of BOLD signal change in the left STS and the left AI reflecting the effect of noise (“Noise > NoNoise”) in the Chew and NoChew conditions. Error bars indicate the standard error of percent signal change (±SEM). To see more clearly the activations in insula, regions illustrated here used a voxel level threshold of *p*<0.005 (uncorrected) and a extent threshold of 200 contiguous voxels.

We first sought brain areas in which blood oxygenation level-dependent (BOLD) responses were higher in Noise than in NoNoise conditions (Figure 3A, Table 1). Noise stimuli elicited higher activations in the bilateral STS, the left anterior insula (AI), and the bilateral inferior frontal gyrus, while the reversed contrast did not show anything significant. Not surprisingly, compared with NoChew condition, chewing gum elicited very strong activations in primary motor area (M1), supplementary motor area (SMA), cerebellum, and Thalamus (see Figure S1 in supporting File S1). The interaction between noise presentation and gum chewing took place in the bilateral STS (Table 1).

**Table 1:** Brain areas revealed by the factorial model.

Regions	BA	Hemisphere	MNI Coordinates			Max *T*-value	Voxel size
			*x*	*y*	*z*		
Noise > NoNoise							
STS	48	L	−50	−12	2	16.31	4703
	48	R	54	−12	−4	15.14	4803
Insula	47	L	−34	22	−2	4.36	463
IFG	45	L	−36	40	14	4.47	323
Chew > NoChew*							
M1 and thalamus		L	−52	−8	28	15.43	4481
		L	−12	−18	2	10.48	
		R	50	−10	34	15.69	5510
		R	14	−18	2	10.64	
SMA		L	−4	−2	60	7.35	302
Cerebellum		L	−14	−64	−24	16.24	1979
		R	18	64	22	15.26	
Chew × Noise							
STS	48	L	−38	−30	10	4.75	296
	48	R	48	−28	8	4.61	335

(STS  =  superior temporal sulcus, IFG  =  inferior frontal gyrus, M1 =  primary motor area, SMA  =  supplementary motor area).

* Since the “Chew > NoChew” contrast revealed very strong activations covering large parts of the brain, and since this contrast is probably most susceptible to motion artifact, we used a more stringent threshold, i.e., *p*<0.05 (FWE) at peak voxel containing more than 30 contiguous voxels.

#### Parametric model

Parametric analysis of functional fMRI data is capable of revealing brain regions in which activation level varies as a function of participants' self-report of stress (i.e., the SVAS-20 scores, see *Methods, fMRI data analysis*). This approach, based on the methods of linear systems analysis, allows a quantitative comparison of the response amplitudes across participants [30]. Here our analysis focused on the BOLD signals for the duration of the whole trials, although similar pattern of effects was obtained when the signals for only the last 10 seconds (i.e., after chewing and without potential head movements) were analyzed (see Figure S2 in supporting File S1). In this statistical model, the first regressor codes the fixed amplitude effect (i.e., the average hemodynamic response, collapsing across 4 conditions). The second regressor is the parametric effect, which codes the variable amplitude effect as a function of the self-report of stress. As expected, increased self-report of stress was associated with increased activation in the bilateral STS (Figure 3B, Table 2). The left AI (MNI coordinates: [−34, 24, −8]; maximum *t* = 3.78, *p*(FWE)  = 0.027) also showed a linear relationship to self-report of stress after small volume correction (see *Methods*, *fMRI data analysis*), which centered at the coordinates of the left AI identified in the factorial contrast (MNI coordinates: [−34, 26, −2]). Thus the activations in the bilateral STS and in the left AI can reflect participants' perceived level of stress.

**Table 2:** Brain areas revealed by the parametric model.

Regions	BA	Hemisphere	MNI Coordinates			Max *T*-value	Voxel size
			*x*	*y*	*z*		
STS	48	L	−46	−14	−6	12.66	3418
	48	R	52	−12	0	11.01	4005
IFG (orbitalis)	47	L	−46	46	−6	7.19	632
IFG (opercularis)	48	L	−54	16	24	5.93	516
IFG (triangularis)	45	R	54	24	10	5.58	443
MOG	18	R	30	−92	2	5.78	253

(STS  =  superior temporal sulcus, MOG  =  middle occipital gyrus, IFG  =  inferior frontal gyrus).

We extracted BOLD signals from the bilateral STS and the left AI to test more clearly how gum chewing has any impact on the activations in these areas (see *Methods, fMRI data analysis*). The interaction between Chew and Noise was significant for the left AI, *F*(1, 15)  = 5.05, *p*<0.05, and for the left STS, *F*(1, 15)  = 15.72, *p*<0.005, with the noise-induced activation increase being lower in the Chew conditions than in the NoChew conditions. Figures 3C and 3D depict the time course of BOLD signal change (Noise > NoNoise, difference wave) in the Chew and NoChew conditions for the left STS and the left AI. Clearly, the noise-induced activation was significantly attenuated in the Chew condition as compared with in the NoChew condition.

### Psychophysiological interaction (PPI)

**Figure 4:**
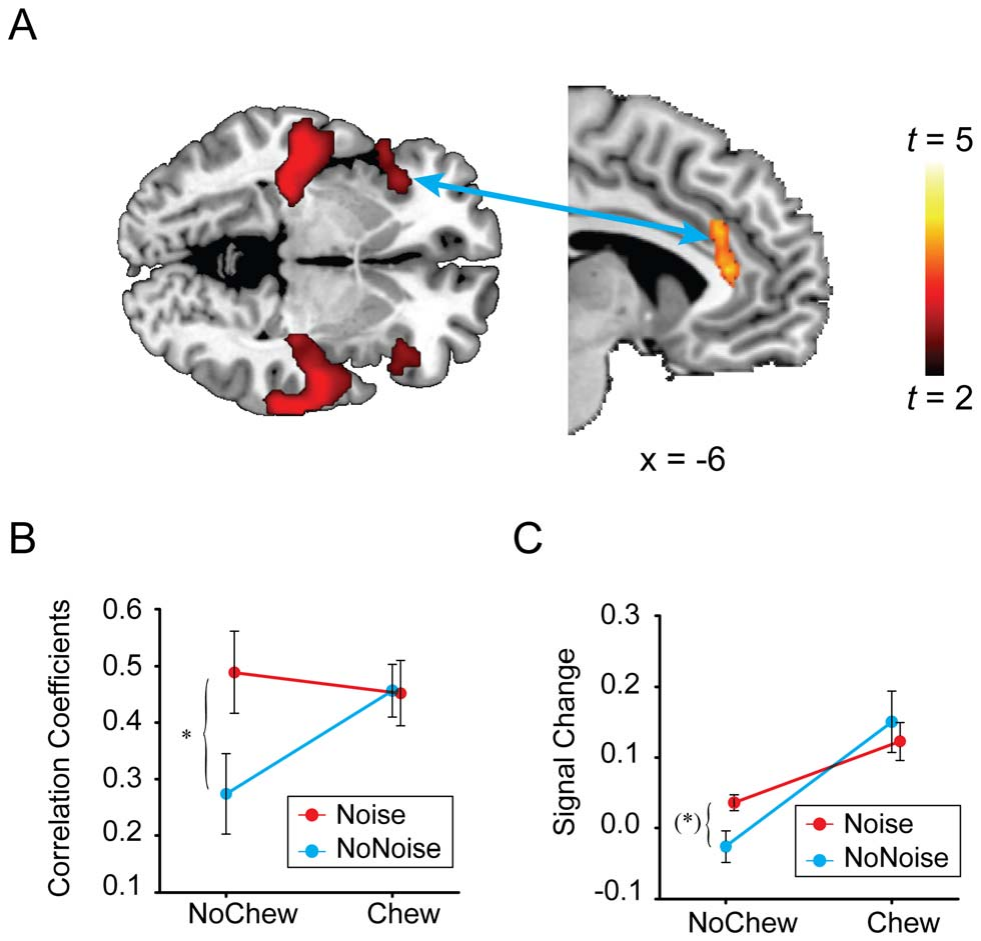
Results of the psychophysiological interactions (PPI) analysis with a left AI seed. (A) The dACC demonstrated larger increase in functional connectivity with the left AI in the NoChew conditions (NoChew_Noise > NoChew_NoNoise) than in the Chew conditions (Chew_Noise > Chew_NoNoise). (B) The correlation coefficients between the BOLD signal in the left AI seed and that in the dACC (for detail, see *Method: Psychophysiological interaction*). (C) The BOLD signal extracted from the dACC in (A). Error bars indicate the variance of the correlation coefficents (±SEM). * *p*<0.05; (*) 0.05<*p*<0.07.

We conducted PPI analysis to find brain regions in which functional connectivity (“Noise > NoNoise”) with the left AI was modulated by gum chewing (see *Methods, fMRI data analysis*). The contrast in functional connectivity between NoChew and Chew conditions revealed activation in the dACC (MNI coordinates: [−6, 34, 12], maximum *t* = 4.18, cluster size: 188 voxels; Figure 4A). To illustrate the connectivity between the left AI and the dACC, we plotted the correlation coefficients of the signal change extracted from these areas for each condition (Figure 4B). Repeated-measures ANOVA on the correlation coefficients showed that the interaction of Chew and Noise was significant for the connectivity between the left AI and the dACC, *F*(1, 15)  = 5.05, *p*<0.05, which was mainly driven by the significant difference between the NoChew_NoNoise and NoChew_Noise conditions, *t*(15)  = 2.34, *p*<0.05. The correlation coefficients in the Chew conditions did not differ significantly.

Indeed, if we plot the signal changes at the dACC, as identified in the above PPI analysis, we found a similar interaction of BOLD signals between noise presentation and gum chewing (Figure 4C).

### Dynamic causality modeling (DCM)

**Figure 5:**
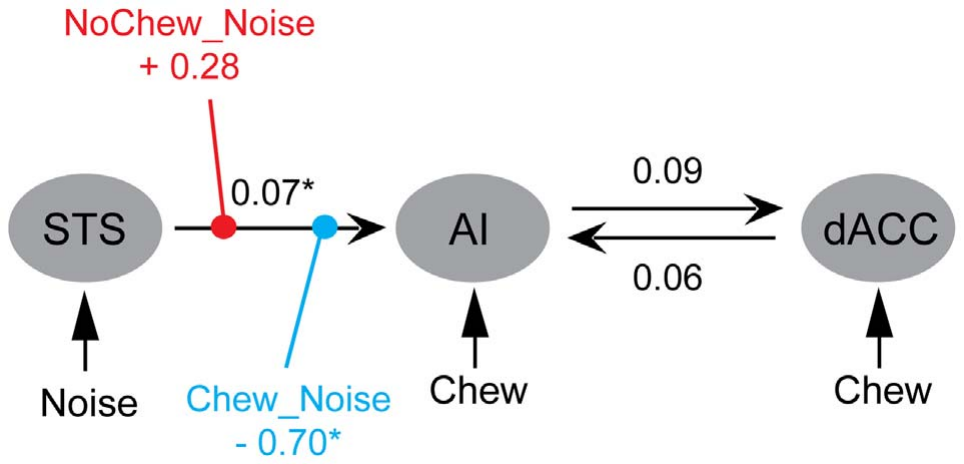
The structure and parameters of the model with the best fit (Model 1). The black lines illustrated the intrinsic connectivities between brain regions. Intrinsic connectivity refers to the connectivity between regions across the whole scanning session, irrespective of stimulus and task. In color are the modulations of stimulus/task on the intrinsic connectivities. The numbers are the strength of connectivity (Hz). * *p*<0.05, corrected for multiple comparison with Bonferroni's procedure.

To investigate at what stage gum chewing influences the information flow in the stress network identified above, we conducted DCM analyses on the network consisting of the left STS, the left AI and the dACC. Bayesian model selection (BMS, [31]) favored the model in which the external perturbations exert the modulatory effect on the projection from the left STS to the left AI (see *Method*, *fMRI data acquisition and analysis* and Figure S3 in supporting File S1). Figure 5 depicts the connectivity parameters based on the winning model (also see Table 3). The modulation of the Chew_Noise condition on the connectivity from the left STS to the left AI (−0.70±0.90 Hz) was significantly less than zero, *t*(15)  = −3.13, *p*<0.007, and significantly less than the modulation of the NoChew_Noise condition (0.28±0.69 Hz), *t*(15)  = −4.46, *p*<0.005. This indicated that while the NoChew_Noise tends to enhance the connectivity from the STS to the AI, the Chew_Noise inhibits that connectivity.

**Table 3:** Parameters of the model with the best fit (Model 1), including inputs, intrinsic connectivities, and modulations of the intrinsic connectivities.

Parameter	Mean ± SD (Hz)
Intrinsic	
lSTS -> left AI	0.07±0.10*
left AI -> dACC	0.09±0.24
dACC-> left AI	0.06±0.16
Modulatory (on lSTS -> left AI)	
NoChew_Noise:	0.28±0.69
Chew_Noise:	−0.70±0.90*
Input	
NoChew_Noise to lSTS	−0.01±0.04
Chew_Noise to lSTS	−0.05±0.05**
Chew_Noise to dACC	−0.02±0.03*
Chew_Noise to left AI	−0.02±0.07
Chew_NoNoise to dACC	0.01±0.08
Chew_NoNoise to left AI	**0.05±0.07**†

(* *p*<0.05, ** *p*<0.01, corrected for multiple comparison following Bonferroni's procedure; † *p*<0.05, uncorrected).

## Discussion

In this study, we investigated the neural effects that gum chewing, as a stress reducer, on noise-induced stress. The participants' rating of stress during fMRI scanning showed that the noise stimuli were effective in inducing stress and that gum chewing was able to reduce the level of this noise-induced stress. In a separate experiment with the same experimental conditions as this study, we recorded participants' skin conductance level (SCL), which is a valid physiological index of stress [32], [33]. The noise effectively caused an increase in participant's self-reported stress and SCL levels, but this increase was lower when participants were simultaneously chewing gum (see Figures S4 and S5 and supporting File S1). The fMRI results showed that gum chewing relieves stress by both attenuating the sensory processing of external stressors and by inhibiting the propagation of stress-related information in the brain stress network.

Gum chewing reduced the activation in the bilateral STS, which was higher in Noise than in NoNoise conditions and which tracked participants' self-reported stress. This pattern mirrors the interaction between noise presentation and gum chewing for the participants' self-reported stress (i.e., SVAS-20, see Figure 2). A possible interpretation for this reduction of sensory processing is that gum chewing distracts participants' attention away from the noise stimuli. Previous neuroimaging studies have demonstrated that performing demanding cognitive tasks (e.g., a 2-back matching task) can attenuate the sensory processing of pain and can reduce participants' painful feeling [34], [35]. The sensory processing in the primary and secondary auditory cortex can also be modulated by top-down attention [36], [37]. Gum chewing or some other activity may shield the organism from the external stressor (i.e., the noise) through attention distraction and reduces the experience of stress. A further elaboration of this account could be that gum chewing generates internal noise within the auditory system which may itself partially mask the impact of external noise or distract attention away from external noise. Whatever the exact process of the functioning of gum chewing, it is clear that gum chewing reduces the subjective or affective responses to the external noise.

The anterior insula (AI) plays a central role in integrating interoceptive and affective information and in generating subjective affective experiences [15], [38]. Critchley et al [16], for example, found that the neural activation and the grey matter volume in the AI predicted participants' performance in an interoceptive task (e.g., heartbeat detection task) and their subjective ratings of visceral awareness. Our finding that the activation in the AI tracked participants' self-reported stress is thus consistent with the interoceptive view of insular function. Critically, we found that gum chewing decreased the activation of the AI, consistent with the finding that active emotion regulation strategies (e.g., reappraisal) down regulate the insular activation for aversive visual stimuli [39]. Although insula activation could be driven by auditory input *per se*, two reasons allow us to argue that the insula activation observed here reflected, at least partially, the participant's experience of stress. First, as revealed by the parametric analysis (Figure 3B), the insula activation positively correlated with participant's rating score of stress in a trial-wise manner while the physical properties of the auditory stimuli remained unchanged. Second, the insula has been implicated in representing psychological stress in previous neuroimaging studies that adopted non-auditory stressors [10], demonstrating the general functions of insula in face of stress.

The dACC is conventionally viewed as responsible for attention and executive control, particularly important for adjusting the physiological and mental states of the organism in preparation for potential challenges [19], [40]. Recently, growing evidence extends the role of dACC to emotional appraisal associated with bodily reactivity and subjective distress [41], [42]. The dACC is found to functionally interact with the AI in many behaviors [15], [18], [43]. Gianaros et al [9], for example, found that the dACC is functionally more connected to the right insula in stressful situations, as compared with less stressful situations. The authors interpreted this strengthened connectivity as reflecting the generation, representation, and control of autonomic activity. The dACC and the AI “have a close functional relationship, such that they may be considered together as input and output regions of a functional system … for regulating physical states and for generating subjective experiences (feelings) on the basis of those states” [42]. Consistent with this view, we found that the functional connectivity between the dACC and the left AI was increased in the NoChew_Noise condition, as compared with the NoChew_NoNoise condition, reflecting an increased demand for the control of the autonomic responses to the noise.

It is important to note that although gum chewing by itself increased the functional connectivity between the dACC and the left AI, the simultaneous presence of noise and chewing gum did not increase this connectivity further (Figure 4B). In line with this, the BOLD activation in the left AI and in the dACC showed a similar pattern as that for the AI-dACC connectivity (see Figures 4C and S6). Since chewing can generate internal noise in the brain [44], an interpretation of the functional connectivity pattern observed here could be that the self-produced noise during chewing perturbed the functioning of the auditory and the stress-related systems so that the external stressor (i.e., external noise) could no longer produce any additional effect. On the other hand, the effect of self-produced noise could be canceled out within the neural system [45], [46] and does not lead to increased stress. In this way we can interpret this interaction between gum chewing and noise in the connectivity as demonstrating that gum chewing lowers the demand for regulation and control for the *external* stressor. Consistently, our DCM analysis showed that gum chewing reduces the propagation of the sensory information from the left STS to the left AI, indicating that gum chewing attenuates the interoceptive processing through which the sensory information of the external stressor is transformed to conscious feeling of stress. In line with our DCM result that gum chewing reduced the effective connectivity from the STS to the AI, the effect of chewing on noise-induced BOLD signal increase seemed to emerge earlier in the STS than in the insula (Figures 3C and 3D). This finding indicates a grade in information flow from the sensory to the interoceptive system. However, the temporal sluggishness of BOLD signal makes it hard to say anything decisive.

The bilateral inferior frontal gyrus (IFG) showed up both in the factorial and the parametric analyses, indicating its role in stress processing. The IFG was found to respond to psychological stress induced by recalling anxiety-provoking personal episode [47], or by demanding cognitive task [48]. Moreover, the dysfunction of IFG in responding to negative stimuli (e.g., trauma) was proposed to be a neural marker of Posttraumatic Stress Disorder [49]. Our finding that the activation level of IFG positively correlated with participant's subjective experience of stress confirmed the role of IFG in representing stress.

Several limitations of the current study should be noted. First, certain brain regions other than the insula and the IFG are found to be important in stress processing, including particularly the amygdala, the midbrain (periaqueductal gray, PAG), and the orbitofrontal cortex [50], [51]. A survey of literature revealed that the amygdala is more likely to show up in the paradigm in which fear-related acute stressor were used [10], [52], [53]. Since we did not use fear-related stimuli (e.g., fearful faces or movies) in our experiment, it is conceivable that the amygdala did not show up even at a liberal threshold (*p*<0.005, voxel level, uncorrected). However, for the midbrain PAG and the orbitofrontal cortex, we did observe significant effect of Noise when we use 150 voxels as the extent threshold (*p*<0.001, voxel level, uncorrected). Specifically, the midbrain PAG was more activated during noise presentation (peak coordinates: [−12, −20, −10]; *t* = 5.99; 159 voxels) whereas the orbitofrontal cortex showed a deactivation in response to noise (peak coordinates: [−6, 36, −24]; *t* = 3.80; 174 voxels). The activation of these regions, however, was not modulated by gum chewing, perhaps due to the low signal-to-noise ratio in these parts of the brain.

Second, the auditory stimuli we used in this study could drive the STS and the AI activation without making the participant feel stressful [54]. It could be more compelling for future studies to investigate stress response of these regions using stimuli other than noise. Nonetheless, we do believe that the activation of the AI reflected, at least partially, the participant's experience of stress, for two reasons. First, as revealed by the parametric analysis (Figure 3B), the insula activation positively correlated with participant's rating score for stress in a trial-wise manner. Second, the insula has been implicated in representing psychological stress by previous neuroimaging studies that adopted non-auditory stressor [10].

It should also be noted that we do not claim that any conclusions derived from this study are specific to gum chewing or to any sub-component of gum chewing (e.g., mastication or taste). Indeed, other actions, such as food eating, may also contribute to stress reduction [29]. We intended to elucidate how stress coping is achieved in the brain by a particular activity (i.e., gum chewing). The reason for using gum chewing is twofold: on the one hand, gum chewing is widely adopted in some cultures as an easily accessible venue of stress reduction [23]; on the other hand, the anti-stress properties of gum chewing [24]–[27] can be used as a model for investigating the neural basis of stress coping and emotion regulation in general.

To conclude, by using fMRI, we identified a neural network for processing noise-induced stress, which consists of sensory, interoceptive, and control modules. Noise increases both the activity and the interconnectivity within this stress network. Gum chewing counteracts the effect of noise on the activity of each module and the functional connectivity between them. Specifically, gum chewing relieves stress by attenuating the sensory processing of external stressor and by inhibiting the propagation of stress-related information in the brain stress network.

## Methods

### Participants

Twenty-four healthy right-handed graduate and undergraduate students took part in the fMRI scanning. Because of excessive head movements (>3 mm), 8 were excluded from data analysis, leaving 16 participants (age 22.7±1.8 yrs, 6 female) in the final set. None of the participants reported any history of psychiatric, neurological or cognitive disorders. Consent was obtained from each participant before scanning. The study was carried out in accordance with the Declaration of Helsinki and was approved by the Ethics Committee of the Department of Psychology, Peking University.

### Design and Stimuli

The experiment used a 2*2 factorial design, with gum chewing (Chew vs. NoChew) and noise presentation (Noise vs. NoNoise) as two within-participant factors. Thirty-two pieces of noise stimuli, selected according to their capacity for inducing stress, were used in the SCL study and the fMRI study (see supporting File S1).

### Procedures

On the day before the fMRI scanning, participants were familiarized with the experimental procedure. They were asked to lie on a bed and simultaneously chew a gum while listening to 15 pieces of noise stimuli not used in the scanning session. During this process, the participants were encouraged to minimize their head movement while chewing. They were also trained to use the computer-version of the SVAS. On the scanning day, each participant was offered a piece of gum of his/her choice. A written instruction “No chewing” was presented under the central fixation sign during the whole experiment except during the chewing phase of a trial in the Chew condition (Figure 1). For each trial, the participant saw first this instruction and the fixation sign for 4 s. To measure the participant's baseline stress level, a computer version of the SVAS scale, a horizontal line with a moving cursor on it, was presented at the onset of the 5th second at the center of the screen, replacing the fixation sign (i.e., the SVAS-5). Participants rated their stress level from 0 to 100 by stopping a moving cursor on a horizontal scale; the initial direction of the cursor's movement was balanced across conditions to remove any effect of sensorimotor confounds. The SVAS scale was presented for 3 s, followed by the fixation sign for 12 s. During this period, a written instruction was presented with the fixation sign; for the Chew conditions, the instruction was “Keep chewing,” which prompted the participant to continue chewing as long as the instruction remained on the screen; for the NoChew conditions, the instruction was “No chewing”; for the Noise conditions, in addition to the visual instruction, a noise stimulus was presented for 10 s from the beginning of the 10th second. For all the conditions, at the beginning of the 20th second, another SVAS scale was presented and the participants were asked to indicate their current level of stress (i.e., the SVAS-20) within 3 s. Finally, the fixation sign and the instruction of “No chewing” were presented again for 7 to 9 seconds. Each full trial lasted for 29 to 31 seconds and the participant was asked to fixate on the fixation sign throughout the trial. The scanning session contained 64 trials (16 per condition) and lasted about 32 minutes. Participants viewed the screen through an angled mirror on the head-coil. Auditory stimuli were presented via an MRI-compatible headphone.

### fMRI data acquisition and analysis

A Siemens 3T Trio scanner with a standard head coil at the Beijing MRI Center for Brain Research was used to obtain T2*-weighted echo-planar images (EPI) with blood oxygenation level-dependent (BOLD) contrast (matrix, 64×64, in-plane resolution, 3 mm×3 mm). Thirty-seven transversal slices of that covered the whole brain were acquired according to an interleaved order with a 0.4 mm gap (repetition time: 2200 ms, echo time: 30 ms, field of view: 220 mm * 220 mm, flip angle: 90°, matrix size: 64*64, voxel size: 3.4 mm * 3.4 mm * 3.5 mm).

The obtained fMRI data were preprocessed and analyzed using Statistical Parametric Mapping software SPM8 (Wellcome Trust Department of Cognitive Neurology, London, UK). The first five volumes of each session were discarded to allow stabilization of magnetization. Preprocessing was done with SPM8 default settings. All images were transformed into standard MNI space and re-sampled to 2×2×2 mm^3^ isotropic voxel. The data were then smoothed with a Gaussian kernel of 8 mm full-width half-maximum to accommodate inter-subject anatomical variability.

### Analyses on BOLD activation

Statistical analyses based on GLM were performed first at the participant level and then at the group level. Each trial was modeled as a boxcar function spanning the whole trial convolved with a canonical hemodynamic response function (HRF). We carried out two model-based analyses. For the factorial model, the four conditions were modeled with separate regressors. For the parametric model, all trials were included in a single regressor, accompanied by a parametric regressor containing the self-report of stress (i.e., SVAS-20) in each trial. The six rigid body parameters were also included in both models to account for head motion artifact. At the group level, a flexible factorial design was used for the factorial model and a one-sample *t*-test was used for the parametric model according to the nature of the design matrix. For the whole-brain exploratory analysis, activation foci survived the threshold of *p*<0.001 uncorrected at peak voxel level and cluster extent threshold *P*<0.05 (FWE). Since the left AI activation in the parametric analysis did not reach the above activation threshold, but this activation was obvious in the factorial analysis, we conducted a region-of-interest (ROI) analysis within the left AI, as identified in the factorial analysis, using the small volume correction approach. Activation within the left AI ROI survived *p*<0.05 (FWE) at peak voxel level.

Signal change data were extracted with the eigenvariable function in SPM8. ROIs were defined as a 4 mm-radium sphere centered at the peak voxel. The eigenvarible of the ROI was a weighted-average of the percent signal change of all the voxels inside the ROI. After extraction, the timeseries was detrended and averaged in an event-related manner.

### Psychophysiological interaction (PPI)

To investigate how gum chewing modulates the functional connectivity between the identified left AI and the rest of the brain, we performed a psychophysiological interaction analysis (PPI, [55]). We computed a PPI map with the contrast “Chew_Noise > Chew_NoNoise” and another with the contrast “NoChew_Noise > NoChew_NoNoise”. We then contrasted the former with the latter using the two-sample *t*-test in SPM8. This contrast was supposed to reveal the brain regions in which the functional connectivity (Noise minus NoNoise) with the left AI was reduced by gum chewing. Activation foci surviving the threshold of *p*<0.001 (uncorrected) at voxel level and *p*<0.05 (FWE-corrected) at cluster level were reported. To display the details of the changes in coupling between seed regions and the significant activation foci derived from PPI analysis, we computed the cross-region signal correlation (Pearson) based on the regional percent signal change timecourse. Specifically, the regional BOLD signal change, the mean of the eight sampling points following the onset of noise presentation (or the corresponding time period in the NoNoise conditions), was extracted. The correlation coefficients between the signal changes in the seed (left AI) and the target (dACC) were calculated for each condition.

### Dynamic causality modeling (DCM)

Bilinear DCM was used in this study [56]. The three activation time courses were extracted from the left STS, the left AI and the dACC ROIs in each participant from a 4-mm sphere centered on the group peak. The left STS and the left AI were identified by the parametric analysis; the dACC was identified by the PPI analysis. We constructed six models that shared identical intrinsic connectivity pattern but varied in terms of the modulatory connectivity (Figure S3 in supporting File S1). The six models were compared using random-effect Bayesian Model Selection (BMS, [31]), by which the “exceedance probability” (the probability of each model being more likely than any other model) of each model was calculated.

## Supporting Information

File S1 **File S1 contains Supplemental Methods, Supplemental Results and Supplemental Figures.**
(DOC)
