# Supplementary material for: Gum Chewing Inhibits the Sensory Processing and the Propagation of Stress-Related Information in a Brain Network
Source: PLoS One. 2013 Apr 3;8(4):e57111. doi: 10.1371/journal.pone.0057111 (PMC3616056; doi:10.1371/journal.pone.0057111)
Supplement: File S1 — contains Supplemental Methods, Supplemental Results and Supplemental Figures. (DOC) [file pone.0057111.s001.doc]

*Supplementary information for*

Gum chewing inhibits the sensory processing and the propagation

of stress-related information in a brain network

*Hongbo Yu^1^, Xi Chen^1^, Jinting Liu^1^, Xiaolin Zhou^1,2^*

**1** Center for Brain and Cognitive Sciences and Department of Psychology, Peking University, Beijing 100871, China

**2** Key Laboratory of Machine Perception (Ministry of Education), Peking University, Beijing 100871, China

**Supplement Methods**

**Stimuli**

Eight categories of sound materials collected from internet were used for generating stressful noise, including the sound of siren, alarm, crash, traffic, gun shooting, etc. Each piece of noise stimulus, lasting for 10 seconds, was created by superimposing three or more different sound samples from a particular category. Twenty-four pieces of stimuli were generated and were tested for their efficacy in inducing stress in a pilot study. In this study, 16 participants, who did not participate in the fMRI or the skin conductance (see below) experiment, were presented with all the 24 pieces of noise stimuli. They were asked to rate, at the end of each piece, their experienced stress on a visual analog scale (0 to 100, with 100 indicating unbearably stressful) presented on the screen. Their skin conduce level (SCL) was simultaneously recorded with the presentation of noise. Eight pieces were discarded due to their relatively poor capacity for inducing stress (rating: 36.0±3.5; SCL: 0.02±0.56 μS) relative to the remaining 16 pieces (rating: 38.6±4.0; SCL: 0.44±0.51 μS). We then created a temporally reversed version of stimuli based on the original 16 remaining pieces of noise. The stimuli of the reversed version were thus matched to the original ones on overall spectral content, intensity, duration, and acoustic complexity. We tested the validity of all the 32 stimuli in a separate SCL experiment serving as a pilot study for the fMRI scanning (see below). In that pilot experiment, as in the fMRI study, the original and the reversed stimuli did not differ in terms of the capacity for inducing the subjective feeling of stress and SCL (all *F*s < 1).

The loudness of these stimuli ranges from 73 to 80 dB in the pilot SCL study and was about 95 dB in the fMRI experiment. However, the participants wore a pair of earplug to protect their hearing. For each category, two of the four stimuli were used in the Chew condition while the other two were used in the NoChew condition. The mapping between noise piece and the Chew/NoChew condition was counterbalanced across participants.

**The pilot SCL study**

*Participants*

Eighteen healthy (13 female) right-handed university students took part in the pilot SCL study. The mean age of participants was 22.8 years (*SD* = 2.1, ranging from 20 to 27 years). None of the participants reported any history of psychiatric, neurological, or cognitive disorders. Consent was obtained from each participant. The experiment was carried out in accordance with the Declaration of Helsinki and was approved by the Ethics Committee of the Department of Psychology, Peking University.

*Data acquisition and analysis*

Participants were seated comfortably and were asked to sit still. Psychophysiological data were recorded using an MP150 psychophysiological monitoring system (BioPac Systems, Santa Barbara, California). Skin conductance was recorded using Ag/AgCl electrodes filled with isotonic NaCl unibase electrolyte, attached to the volar surface of the second phalanx of the index and ring fingers of the left hand. Room temperature was maintained at 25 – 27 degree centigrade. SCL was calculated by averaging the skin conductance signal across each trial. We carried out a 2 (Chew vs. NoChew) × 2 (Noise vs. NoNoise) repeated-measures ANOVA for the SCL change relative to baseline (i.e., the mean SCL during the 5 s-period prior to the onset of each trial).

**Supplement Results**

**Behavioral results in the pilot SCL study and the fMRI study.**

We carried out 2 (Chew vs. NoChew) × 2 (Noise vs. NoNoise) repeated-measures ANOVA for the SVAS-5 ratings and for the SVAS-20 ratings, respectively. For the SVAS-5 rating, no effect reached significance, indicating that the baseline stress state was equivalent across the four conditions. For the SVAS-20 rating, both main effects and the interaction were significant.

For the SCL study (Figure S5), the ratings were higher in the Noise conditions (64±22) than in the NoNoise conditions (20±14), *F* (1,17) = 57.66, *P* < 0.001, and higher in the NoChew conditions (45±14) than in the Chew conditions (39±15), *F* (1, 17) = 7.03, *P* < 0.05. The interaction between Chew and Noise was significant, *F* (1, 17) = 4.23, *P* = 0.055, with gum chewing significantly reducing the subjective stress rating in the Noise conditions, *t* (17) = 4.23, *P* < 0.001, but this interaction was not in the NoNoise conditions, (*t* (17) < 1, *P* > 0.1). Comparing Figure S5 with Figure 2, it is clear that the pattern of rating scores in the SCL study (outside of the fMRI scanner) were essentially the same as the one in the scanner.

For the fMRI study (Figure 2), the rating was higher in the Noise conditions (50±17) than in the NoNoise conditions (22±10), *F* (1, 15) = 45.7, *P* < 0.001, and higher in the NoChew conditions (39±13) than in the Chew conditions (33±13), *F* (1, 15)= 5.7, *P* < 0.05. The interaction between Chew and Noise was also significant, *F* (1, 15) = 6.2, *P* < 0.05), such that gum chewing significantly reduced the subjective stress rating in the Noise conditions, *t* (15) = 2.58, *P* < 0.05, but not in the NoNoise conditions, *t* (15) < 1, *P* > 0.1.

**Physiological results in the pilot SCL study**

Both the main effects and the interaction of the SCL were significant (Figure S4). Specifically, the SCL was higher in the Noise conditions (*M* = 0.28 μS, *SD* = 0.36 μS) than in the NoNoise conditions (*M* = -0.08 μS, *SD* = 0.19 μS), *F* (1,17)= 9.96, *P* < 0.01, and higher in the Chew conditions (*M* = 0.25 μS, *SD* = .09 μS) than in the NoChew conditions (*M* = -0.05 μS, *SD* = 0.07 μS), *F* (1,17) = 15.43, *P* < 0.001. Importantly, the interaction between Chew and Noise was significant (*F* (1, 17) = 4.18, *P* = 0.056), such that gum chewing reduced the SCL increase induced by hearing noise. Note that mastication alone can induce large SCL increase. It is thus not surprising that the SCL change was larger in the Chew conditions than in the NoChew conditions.


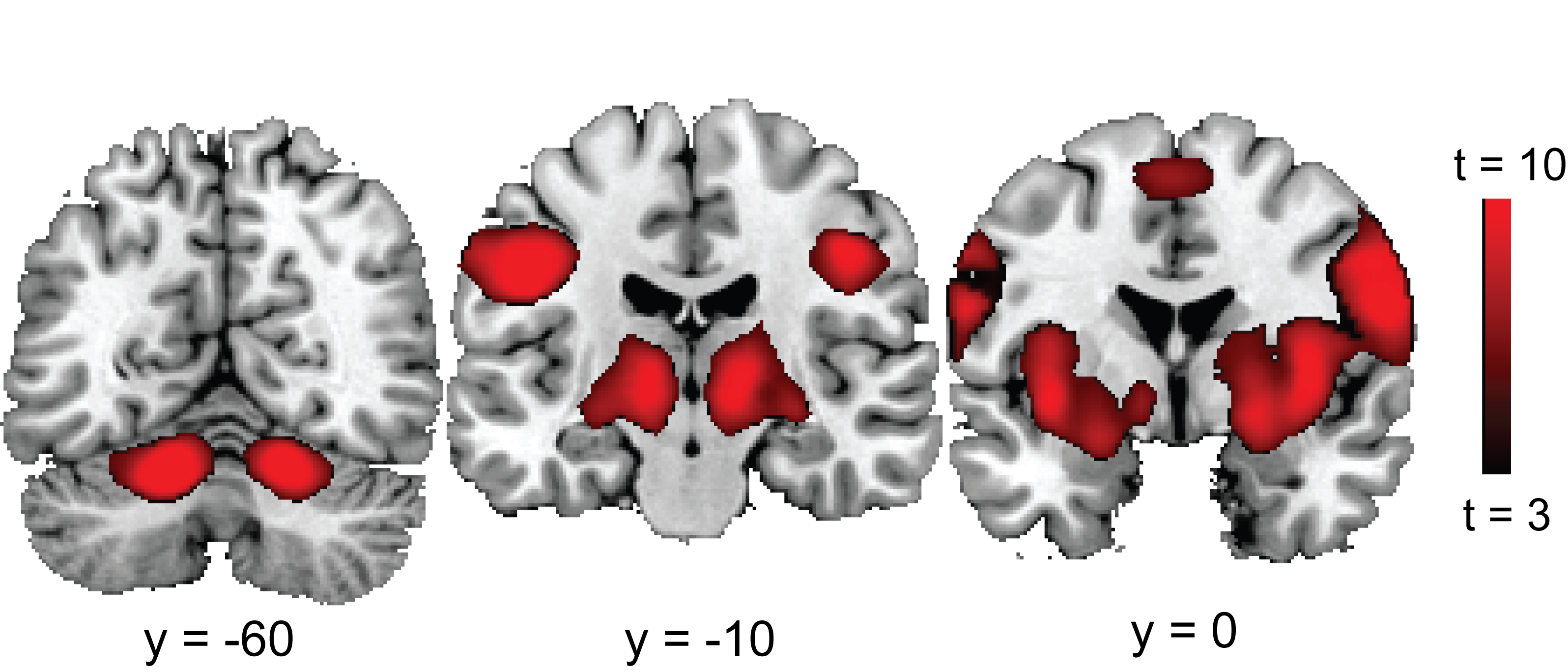


**Figure S1.** The main effect of Chew derived from the factorial model. A more stringent activation threshold was used, with *p* < 0.05 (FWE) for peak voxel and the cluster containing more than 30 contiguous voxels.
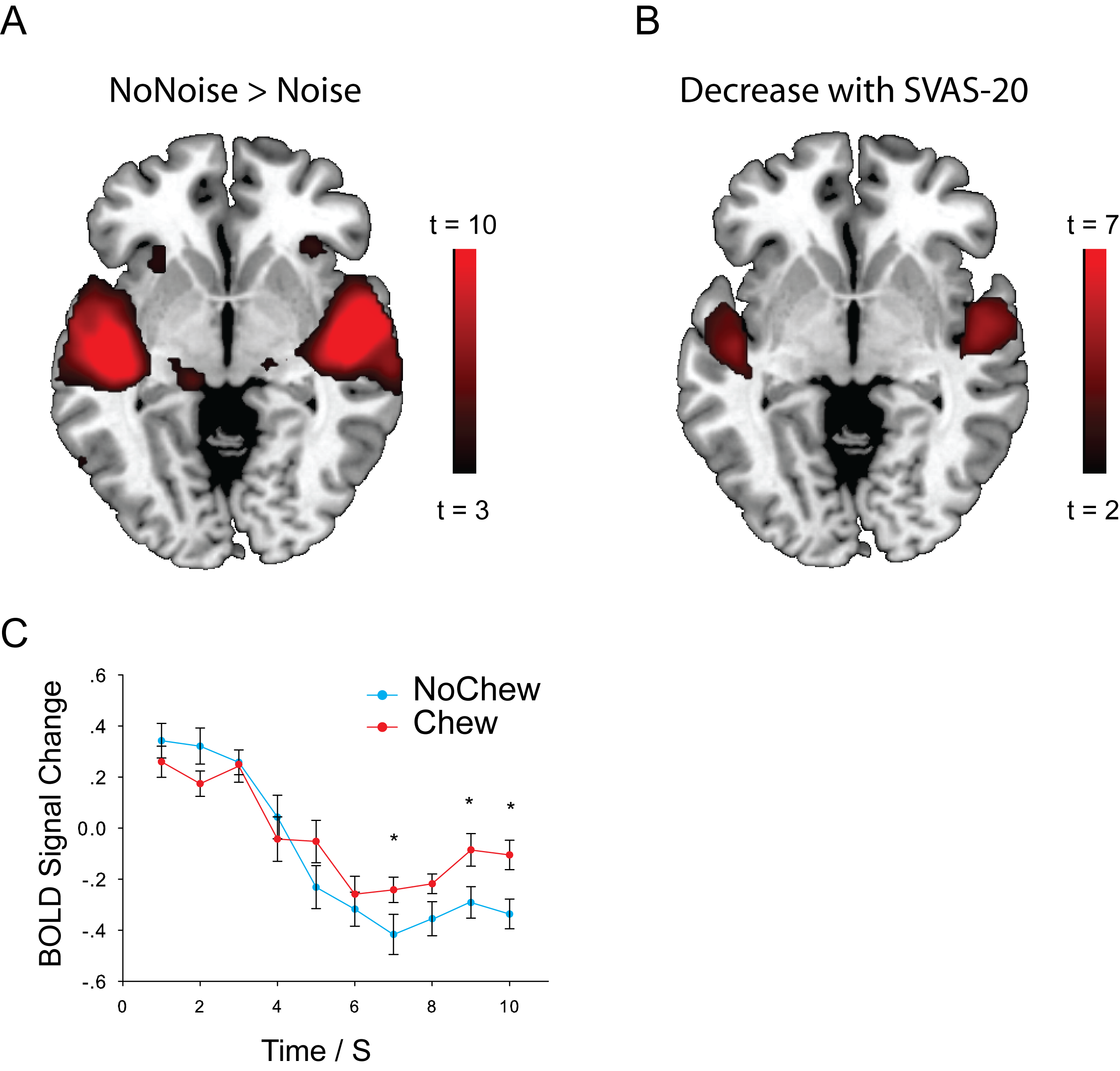


**Figure S2.** Whole-brain analysis (factorial model) was based on the signal from the last 10 seconds of each trial. We utilized the spatial specificity of post-stimulus undershoot, which is the negative BOLD signal following the cessation of stimulation (1-4), to check whether a similar pattern of “deactivation” can be obtained compared with the pattern derived from the analysis of the whole trials. Since the magnitude of the post-stimulus undershoot positively correlates with the magnitude of the stimulus-induced activation, reversed contrasts were defined. The main effect of Noise (“NoNoise > Noise”) (Figure S2A) and the parametric contrast (“Decrease with SVAS-20”) (Figure S2B) showed similar patterns compared with the pattern obtained for the whole trials (Figure 3). The BOLD signal differences between Noise and NoNoise conditions in the left STS extracted from the last 10 seconds of a trial is plotted in Figure S2C. As can be seen, larger deactivation appears in the NoChew conditions relative to the Chew conditions, indicating higher positive activation by noise during stimulus presentation in the NoChew than in the Chew conditions. To see more clearly the activations in insula, regions illustrated here used a threshold with *p* < 0.005 (uncorrected) at peak voxel and cluster containing more than 200 contiguous voxels.


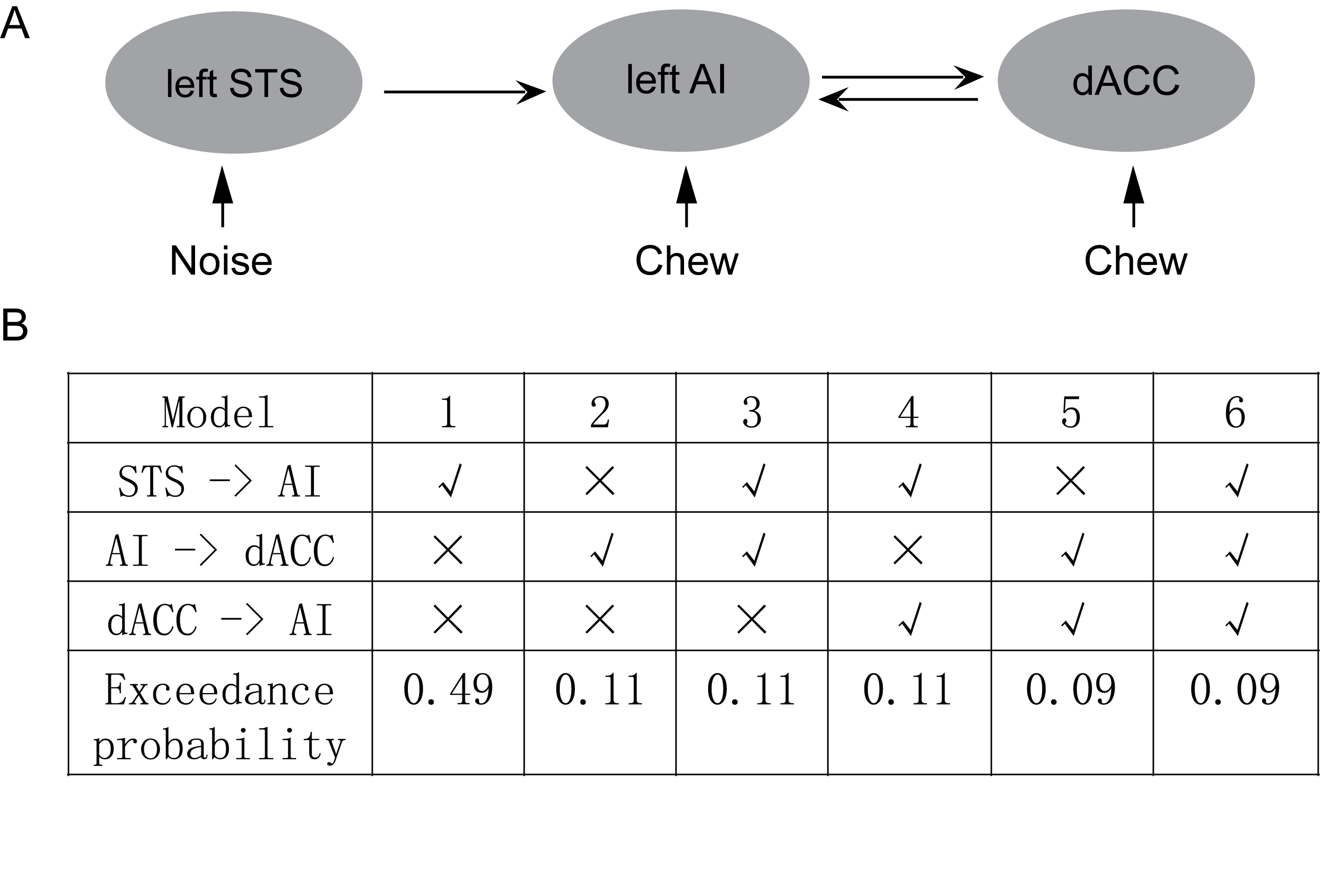


**Figure S3.** DCM Model structure. (A) The intrinsic connectivities were common to all the 6 models. Noise (NoChew_Noise and Chew_Noise) entered the system through the left STS (STS), whereas Chew (Chew_NoNoise and Chew_Noise) affected the activation of the left AI and the dACC. (B) The pattern of modulatory parameters of the 6 models and the corresponding exceedance probability. As can be seen, the first model was strongly favored by Bayesian Model Selection (BMS).


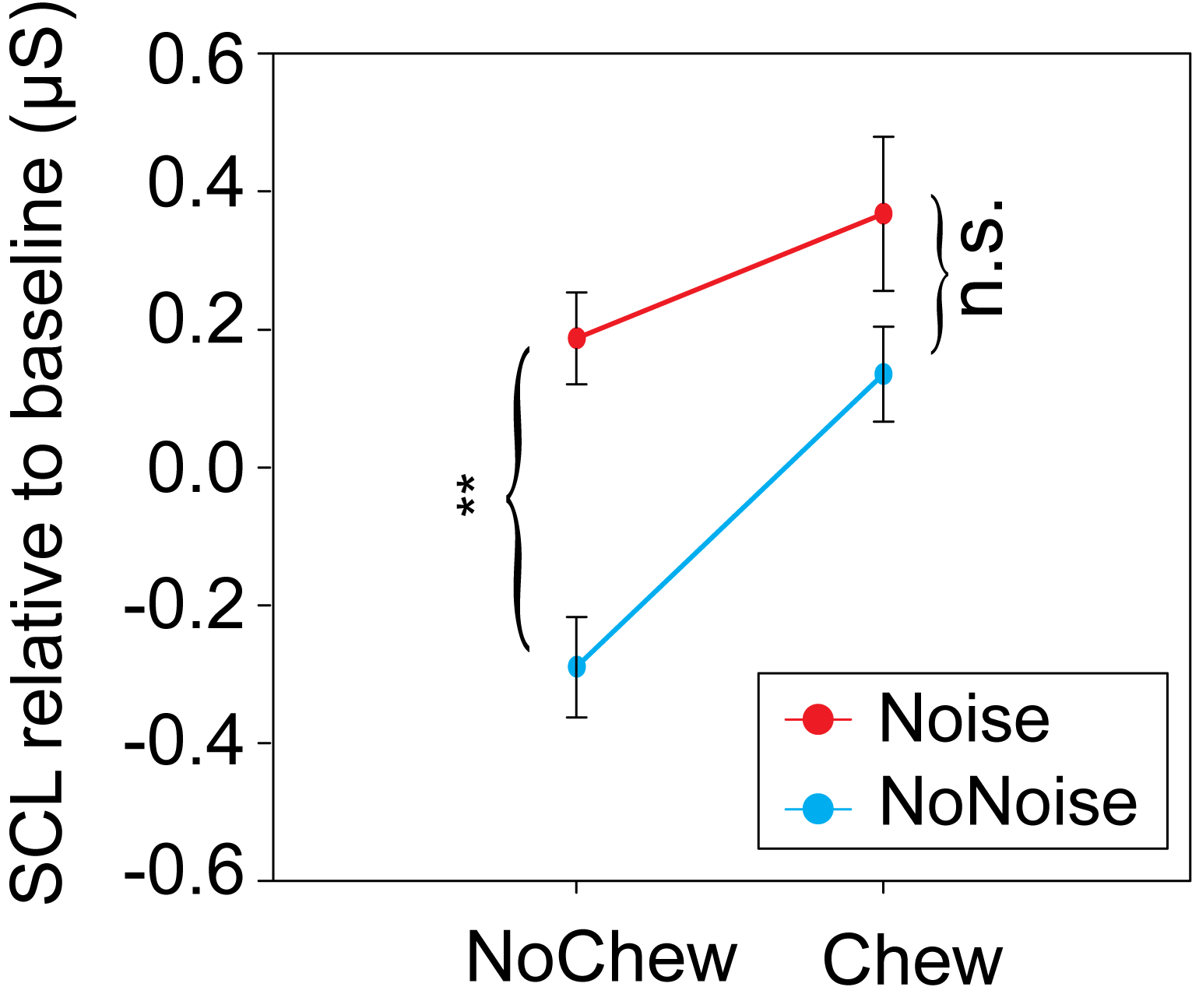


**Figure S4.** The skin conductance level (SCL) results in the pilot SCL study.


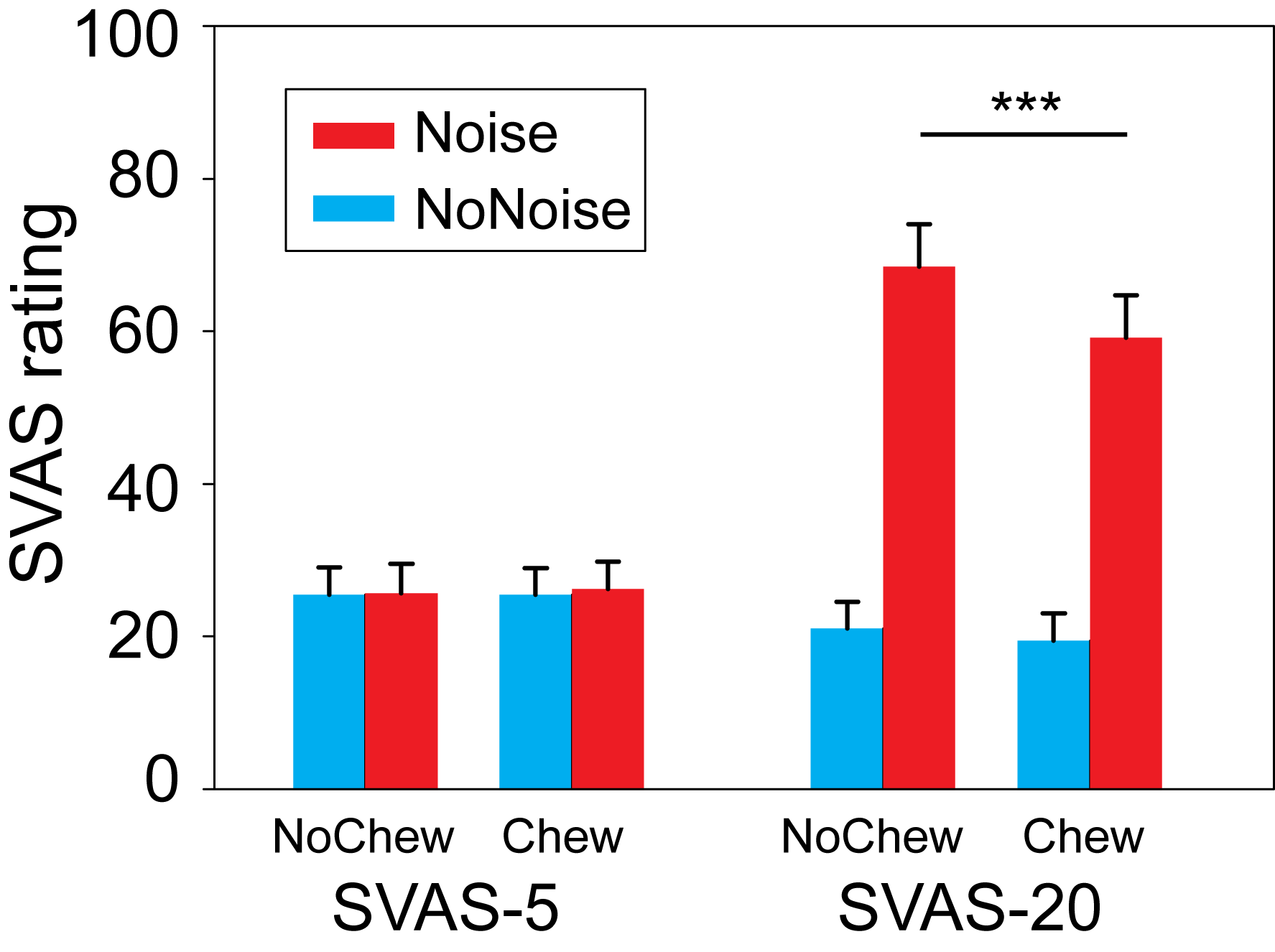


**Figure S5**. Experienced stress before the presentation of noise (SVAS-5, left) and after the presentation of noise (SVAS-20, right) as a function of noise presentation and gum chewing in the pilot SCL study. *** *p* < 0.001


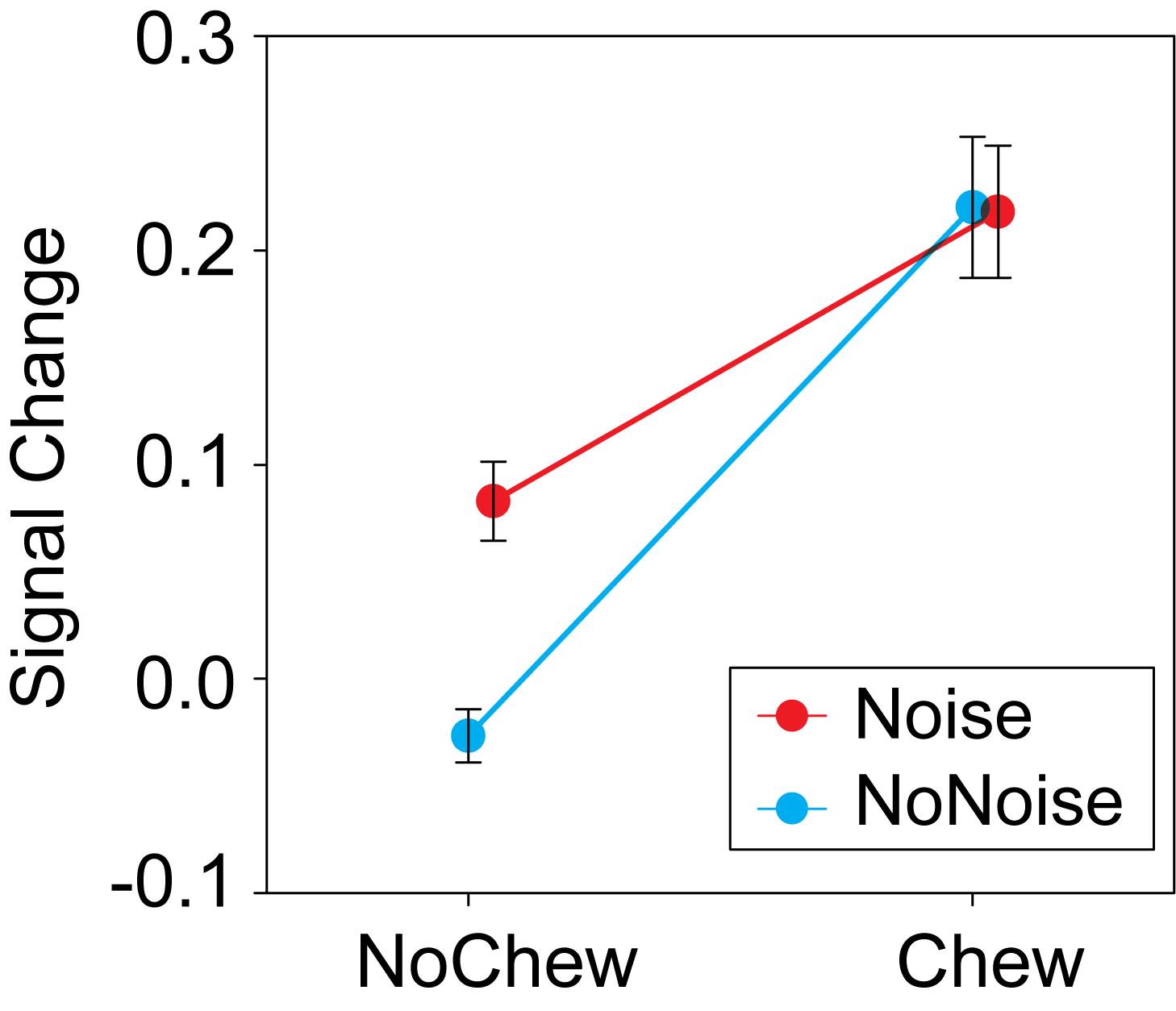


**Figure S6.** The BOLD signal change in the left AI identified by the parametric model.

**Reference List**

1. Kim S, Ogawa S (2012) Biophysical and physiological origins of blood oxygenation level-dependent fMRI signals. J Cereb Blood Flow Metab 32: 1188–1206.

2. Schroeter ML, Kupka T, Mildner T, Uludag K, von Cramon D (2006) Investigating the post-stimulus undershoot of the BOLD signal—a simultaneous fMRI and fNIRS study. NeuroImage 30: 349–358.

3. van Zijl PCM, Hua J, Lu H (2012) The BOLD post-stimulus undershoot, one of the most debated issues in fMRI. NeuroImage 62: 1092–1102.

4. Zhao F, Jin T, Wang P, Kim S (2006) Improved spatial localization of post-stimulus BOLD undershoot relative to positive BOLD. NeuroImage 34:1084–1092.
